# *OGG1 *DNA Repair Gene Polymorphism As a Biomarker of Oxidative and Genotoxic DNA Damage

**DOI:** 10.29252/ibj.25.1.47

**Published:** 2020-08-31

**Authors:** Kanika Miglani, Sunil Kumar, Anita Yadav, Neeraj Aggarwal, Ranjan Gupta

**Affiliations:** 1 Department of Biochemistry, Kurukshetra University Kurukshetra, Haryana 136119, India;; 2 Department of Biotechnology, Kurukshetra University Kurukshrtra, Haryana 136119, India;; 3 Department of Microbiology, Kurukshetra University Kurukshetra, Haryana 136119, India

**Keywords:** 8-hydroxy-2’-deoxyguanosine, 1-hydroxypyrene, Polycyclic aromatic hydrocarbons

## Abstract

**Background:**

Single nucleotide polymorphisms in *OGG1* gene modulates DNA repair capacity and functions as one of the first lines of protective mechanisms against 8-OHdG mutagenicity. *OGG1*-Cys326 gene polymorphism may decrease DNA repair function, causing oxidative stress due to higher oxidative DNA damage. The main purpose of this study was to examine the link of oxidative and genotoxic DNA damage with DNA repair *OGG1 *gene polymorphism, in charcoal workers exposed to polyaromatic hydrocarbons.

**Methods:**

Urinary 8-OHdG excretion (a biomarker of oxidative DNA damage) was determined in both exposed and control populations. Genotyping of *OGG1* DNA repair gene in the blood samples of subjects was carried out by PCR-RFLP method.

**Results:**

The 8-OHdG urinary concentration was significantly higher (*p* < 0.05) in the exposed (geometric mean 12.33 ± 3.78) than in the unexposed (geometric mean 7.36 ± 2.29) population. DNA damage, as measured by 8-OHdG and TM content, was found to be significantly higher in *OGG1* homozygous mutants (mt/mt; 18.81 ± 3.34; 6.04 ± 0.52) as compared to wild-type genotypes (wt/wt; 10.34 ± 2.25; 5.19 ± 2.50) and heterozygous (wt/mt) mutants (12.82 ± 2.81; 6.04 ± 0.93) in the exposed group.

**Conclusion:**

We found a significant association of *OGG1* heterozygous (*wt/mt*) and homozygous (*mt/mt*) variants with oxidative and genotoxic damage, suggesting that these polymorphisms may modulate the effects of PAH exposure in occupational workers.

## INTRODUCTION

During the process of charcoal production, charcoal workers are continuously exposed to wood smoke and charcoal dust containing fine particulate matters such as PAH and certain gaseous pollutants released from burning wood. Therefore, the concern is charcoal workers who do not use any protective equipment, and there is high probability that they are exposed to PAHs during the charcoal production. PAHs are considered as carcinogenic, mutagenic and immunosuppressive agents due to their interference with the normal functioning of cellular membrane and certain membrane-associated enzymes^[^^1^^]^. Exposure to PAHs causes the interaction of this genotoxicant with nuclear DNA, leading to the oxidative and genotoxic DNA damage^[^^2^^]^. 

 An oxidized form of guanosine, 8-OHdG, is a biomarker of oxidative DNA damage and is involved in the pathology of many diseases such as cancer, atherosclerosis, and diabetes^[^^3^^]^. Base excision DNA repair pathway recognizes 8-OHdG as a threatening lesion, which results in its excretion in human urine without further metabolism^[^^4^^]^. Accordingly, estimation of urinary 8-OHdG is increasingly used as a non-invasive biomonitoring approach to assess the oxidative DNA damage produced in the body^[^^5^^]^. Comet assay is applied to assess DNA damage/genotoxicity^[^^6^^]^. Genetic variations in enzyme-encoding genes are involved in xenobiotic metabolism, and DNA repair may influence genotoxicity^[^^7^^]^. The combined use of genetic biomarkers and classic epidemiological tools has enabled the identification of the early effects of occupational exposure to distinct pollutant around the world.

Susceptibility to exogenous and endogenous genotoxicants may be modulated by genetic polymorphisms of DNA repair genes. There are multiple DNA repair pathways for repairing various types of DNA damage. These repair pathways involve a number of proteins that play a role in protecting the genome from mutagenic effects. Single nucleotide polymorphisms in DNA repair genes have been identified^[^^8^^]^ and shown to contribute to alteration in their DNA repair capacity. Therefore, genetic variations in DNA repair genes have been known to modulate the levels of DNA damage in peripheral blood lymphocytes of the workers exposed to PAH at workplace. 

*OGG1* is a DNA repair polymorphic gene involved in the base excision repair pathway. Human *OGG1* is a base excision DNA repair enzyme that participates in the removal of both 8-oxo-dG and 8-oxo-G^[^^9^^,^^10^^]^. Several researchers have already described the association between genetic damage, occupational exposure, and polymorphisms in DNA repair genes, but to the best of our knowledge, it has not been reported in charcoal workers.

## MATERIALS AND METHOD

**Subjects**

The study included 77 individuals working in a charcoal-producing kiln from 16 work stations located in Haryana State, India. Charcoal workers were defined as those involved in charcoal production with working hours more than seven hours per day. The control group was comprised of 79 individuals who were not exposed to PAH generated during charcoal production, but the environmental exposure and socioeconomic status of the control population were matched with the exposed group. Control samples were collected from healthy volunteers of the same area of the exposed population but far from the charcoal-producing kilns. All the subjects enrolled in our study were of Asian origin, living in the rural and suburban area of Haryana. A questionnaire was designed to assess the participants’ demographic characteristics such as age, consumption habits (smoking, tobacco and drinking habits), and years of work exposure. A person who smoked tobacco through cigarette or bidi (more than 10 cigarettes or bidis) in the routine life was considered as a smoker, while the one who never smoked tobacco throughout his life was regarded as non-smoker. In addition, the person who have consumed alcohol in daily life for the past many years, and the one who have never consumed alcohol throughout his life were considered as alcohol and non-alcohol users, respectively.

**Sample collection**

Blood (approximately 5 ml) and urine samples were collected from charcoal workers exposed to wood smoke and control subjects with the help of a trained technician. All the exposed individuals had minimum of two years of work exposure. Blood and urine samples of the subjects were gathered after the charcoal workers finished their work shift.

**Estimation of urinary 1-OHP**

To assess PAH exposure in the exposed population, the level of 1-OHP in the urine samples of exposed and control subjects were analyzed by a former method^[^^11^^]^, through GC/MS with minor modifications. To 2 ml of the urine sample, 10 µl of β-glucuronidase/aryl sulfatase was added, and the sample was incubated at 37 °C for 3 h. The sample was extracted with 5.0 ml of ethyl acetate by mechanical shaking for 20 min. The solution was evaporated in a vacuum rotor to about 0.1 ml. The dry residue was dissolved in 50 µl of *N*-*Methyl*-*N*-(*trimethylsilyl)trifluoroacetamide, *and the tubes were heated at 60 °C for 30 min. Subsequently, a 1-µl sample of the solution was injected into the GC system. The gas chromatograph used was an Agilent (U.S.) 7890 A with a splitless injector. The analytical column was a 30-m HP-5MS column (cross-linked 5% phenylmethyl silicon, 0.2 mm of column internal diameter × 0.25 µm column film thickness. Oven temperature program was set starting at 80 °C, held for 1 min, raised to 320 °C at 20 °C/min and held for 5 min. All mass spectra were obtained with an Agilent 5975 B instrument, and the ion source was operated in the selected ionization mode. Confirmation of the compound was completed by MS characteristic ions, and the ratio of MS characteristic ions and GC retention time was matched with the known standard compound. 

**Estimation of urinary 8-OHdG**

Urinary 8-OHdG was evaluated with a competitive enzymatic immunoassay kit (Caymen chemical Company, Michigan, USA). The concentration of creatinine was measured spectrophotometerically as described before^[^^12^^]^. Level of 8-OHdG was expressed relative to creatinine (ng/mg creatinine).

**Alkaline comet assay**

For genotoxicity assessment, alkaline comet assay was carried out by former methods with minor modifications^[13,14]^. Briefly, dust-free plain slides were covered with a layer of 150 µl of 1% normal melting agarose and dried in a hot air oven for 10 min. The blood samples (5-10 µl) were mixed with 90 µl of warm 0.5% low melting agarose (prepared in phosphate buffer saline), and this mixture was layered as the additional second layer and gelled at 4 °C for 15 min. An additional third layer of 0.5% low melting agarose was added on top and gelled again at 4 °C for 15 min. The slides were then put into freshly prepared, chilled lysis buffer solution at 4 °C for minimum 2 h, followed by incubation in an alkaline electrophoresis buffer for 20 min and electrophoresis (25 V and 300 mA) in the same buffer for 30 min. The slides were then immersed in a neutralization buffer (Tris-buffer) and stained with ethidium bromide (20 µg/ml).

**Comet scoring**

A total of 50 cells from each of the duplicate slides were examined randomly under a fluorescent microscope. The extent of DNA damage was measured quantitatively as TM value using Lucia Comet Assay software (Version 7.12). The TM was defined as the percentage of DNA in the tail multiplied by the length between the centre of the head and tail^[^^15^^]^.

**Isolation of genomic DNA**

The genomic DNA was isolated from the whole blood as described elsewhere^[^^16^^]^. 

***OGG1*** **genotyping**

The presence of the *OGG1* Ser326Cys polymorphism was detected by amplifying genomic DNA with the forward (5’-ACTGTCACTAGTCTCAC CAG-3’) and the reverse (5’-GGAAGGTGCTTGG GGAAT-3’) primers. The 201-bp PCR product was digested with Fnu4H1 at 37 °C for 1 h. Digested products were separated by electrophoresis and visualized by ethidium bromide staining. Wild-type alleles resulted in a 201-bp fragment, while the variant allele resulted in 100 and 101 base pairs following restriction enzyme digestion.

**Statistical analysis**

Mann-Whitney U test was used to compare different variables between the studied groups. The influence of genetic polymorphisms of DNA repair gene and confounding factors on the studied biomarkers among the exposed and control populations was determined by post hoc analysis and Tukey’s HSD test using Multivariate analysis of variance. All tests were performed using statistical software system SPSS 16.0. The level of statistical significance was set at *p* < 0.05.

**Ethical statement**

The above-mentioned sampling protocols were approved by the Human Ethics Committee of Kurukshetra University, Kurukshetra, India (Ethical code: IEC/12/240, 16 may 2012). Written informed consents were provided by all the study subjects.

## RESULTS

**Demographic characteristics**

This study was comprised of 77 PAH-exposed charcoal workers and 79 controls, and their demographic characteristics are shown in Table 1. The number of subjects and the mean age in each age group in both control and exposed workers matched demographically. Charcoal workers included in this study had demographically matched mean age of 34.83 ± 11.05, which ranged from 17 to 57 years old. Regarding consumption habits, i.e. smoking, alcohol consumption, and tobacco chewing, the distribution was found to be non-significant between the charcoal workers and control subjects. The charcoal workers under study were occupationally exposed to wood smoke for an average time of 7.99 ± 4.54 years.

**Estimation of urinary 1-OHP**

In our study, the mean concentrations of urinary 1-OHP levels in both control and exposed subjects were found to be 25.59 ± 16.55 ng/mg creatinine and 161.59 ± 58.77 ng/mg creatinine, respectively. The chromatogram depiction of this result has been reported in one of our published research article^[^^17^^]^.

**Estimation of urinary 8-OHdG**

The 8-OHdG urinary concentration, a marker of oxidative DNA damage, was significantly higher (*p* < 0.05) in the exposed (geometric mean 12.33 ± 3.78; 6.33-24.25) than in the unexposed (geometric mean 7.36 ± 2.29; 3.46-15.20) populations (Table 2).

**Association between oxidative stress repair gene and DNA damage**

The effects of genetic polymorphism of *OGG1* gene on 8-OHdG content in both exposed and non-exposed population were investigated. Genetic variants of *OGG1* in all the subjects were analyzed by PCR-RFLP.

The subjects with the mutant *OGG1* Ser326Cys genotype had significantly higher oxidative DNA damage as measured by urinary 8-OHdG levels, compared to the wild type of *OGG1*Ser326Ser genotype in exposed as well as non-exposed population (*p* < 0.05). The values of 8-OHdG were significantly higher in the exposed population compared to the controls (Table 2). The result for the effect of *OGG1* polymorphism on TM content has been summarized in Table 3. The 8-OHdG and TM content were significantly higher in Cys/Cys *OGG1 *genotypes (mt/mt; 18.81 ± 3.34; 6.04 ± 0.52) as compared to Ser/Cys (wt/mt 12.82 ± 2.81; 5.40 ± 0.84) and Ser/Ser genotypes (wt/wt; 10.34 ± 2.25; 5.19 ± 0.78) in charcoal workers. The levels of both oxidative and genotoxic damage were significantly higher in charcoal workers than in the control population. Results of linear regression analysis adjusted for demographic characteristics such as age, consumption habits, and exposure duration are shown in Table 4.

**Table 1:** Demographic characteristics of the control and exposed groups

**Variables**	**Control ** **(n = 79)**			**Charcoal workers ** **(n = 77)**			**95% CI**		***p*** **value**	**OR**
	**N (%)**		**Mean ± SD**	**N (%)**		**Mean ± SD**	**Lower**	**Upper**		
Age (y) 10-20 21-30 31-40 41-50 51-60	5 (6.32) 21 (26.5) 27 (34.1) 18 (22.7) 8 (3.41)	18.8 ± 0.84 25.57 ± 2.75 32.63 ± 2.51 45.22 ± 2.92 54.38 ± 2.39		7 (9.09) 23 (29.8) 24 (31.6) 16 (20.7) 7 (9.09)	17.7 ± 1.25 25.2 ± 2.84 36.3 ± 2.61 45.5 ± 3.39 54.3 ± 3.20					
Smoking Smoker Non-smoker	46 (58.2) 33 (41.7)			48 (62.3) 29 (37.7)			0.44	1.60	0.99	0.84
Alcohol intake Alcoholic Non-alcoholic	41 (51.9) 38 (48.1)			34 (44.2) 43 (55.8)			0.73	2.56	0.172	1.35
Tobacco chewing Tobacco chewer Non-tobacco chewer	9 (11.4) 70 (88.6)			5 (6.49) 72 (93.5)			0.59	5.80	0.590	1.85
Work exposure (y) <10 10-20 >20				50 (64.9) 25 (32.7) 2 (2.60)	7.99 ± 4.54 5.25 ± 2.24 12.40 ± 1.96 21.50 ± 0.71					

Student’s-*t* test was applied to compare the mean value of age between the control and exposed populations. Chi-square test was applied to test differences in age, consumption habits, and work exposure experience.

## DISCUSSION

Exposure to PAH in charcoal workers can be due to inhalation or by dermal contact. PAH is known to exert toxic effects even at relatively low concentrations. Such toxicity could be modulated by mutant genotypes of DNA repair gene, causing an increase in the susceptibility to various diseases^[^^18^^]^. Thus, the main goal of our study was to assess the modulatory effect of gene polymorphism of DNA repair gene on oxidative DNA damage in charcoal workers. These workers had the higher levels of urinary 1-OHP as compared to reference values, indicating high exposure to PAH. Urinary 1-OHP levels in occupational workers were higher than those of control population as suggested by Wenjuan *et al.*^[^^19^^]^. These observations are also in accordance with those reported previously by Fu *et al.*^[^^20^^]^ and Nguyen *et al.*^[^^21^^]^ who described the toxicity of PAH as a consequence of imbalance between pro-oxidant and antioxidant homeostasis, the so called oxidative stress, which ultimately leads to oxidative damage. Therefore, to assess the differential impact of such toxicants on the exposed individuals, we investigated the association of genetic polymorphism of *OGG1* gene with oxidative and genotoxic damage biomarkers in the exposed and control groups. Our study found that *OGG1* polymorphisms are significantly related to oxidative and genotoxic damage, and mutant type genotype presented higher levels of DNA damage. These results are similar to findings of Chen *et al.*^[^^22^^] ^who showed higher repair activity of *OGG1* Ser/Ser for 8-OHdG than the *OGG1* Cys/Cys. Also, Aka *et al.*^[^^23^^]^ and Pawlowska *et al.*^[^^24^^]^ observed that Cys/Cys and Ser/Cys *OGG1* genotypes had less DNA repair capacity compared to the Ser/Ser *OGG1* genotype.

**Table 2:** Levels of urinary 8-OHdG in the control and exposed populations

**Biomarker of effect**	**Group**	**Mean ± SD**	**Minimum**	**Maximum**	**95% CI for mean**	
					**Lower**	**Upper**
**Urinary 8-OHdG** **(ng/mg creatinine)**	Control	7.36 ± 2.29	3.46	15.20	6.85	7.87
	Exposed	12.33 ± 3.78^*^	6.33	24.25	11.47	13.20

Mann-Whitney U test was applied to compare the mean values between the control and exposed groups. ^*^Significant at* p *< 0.05

**Table 3:** Effect of *OGG1* genotype on urinary 8-OHdG content (ng/mg creatinine) and TM (µm)

**Genotype **	**Control population (n = 79)**			**Charcoal workers (n = 77)**		
	**N**	**Urinary 8-OHdG content (mean ± SD)**	**TM content** **(mean ± SD)**	**N**	**Urinary 8-OHdG content (mean ± SD)**	**TM content** **(mean ± SD)**
**wt/wt**	50	8.15 ± 2.50	1.40 ± 0.33	39	10.34 ± 2.25	5.19 ± 0.78
**wt/mt**	25	6.04 ± 0.93^*^	1.33 ± 0.32	28	12.82 ± 2.81	5.40 ± 0.84
**mt/mt**	4	5.67 ± 0.62^*^	1.01 ± 0.07^*^	10	18.81 ± 3.34^*^	6.04 ± 0.52^*^

Multivariate analysis of covariance test was used to test the differences in the urinary 8-OHdG content; TM was adjusted for age, consumption habits, and exposure duration among the control and exposed groups. ^*^Significant at* p *< 0.05

There are a number of clinical studies in agreement with our results, indicating that the mutant genotypes of *OGG1* have higher DNA damage and lower repair capacity of 8-OHdG than wild-type genotype^[^^25^^,^^26^^]^, which is associated with the risk of lung cancer^[^^26^^]^, type 2 diabetes^[^^27^^]^, breast cancer^[^^28^^]^ and nasopharyngeal carcinoma^[^^29^^]^. Some previous researchers have deduced that the reduced DNA repair capacity of mutant *OGG1* genotypes may result from either the loss of a putative regulatory serine phosphorylation site or the introduction of a redox-sensitive cysteine amino acid at position 326^[^^30^^]^. The process of recognition and repair of 8-oxoguanine by *OGG1* is well understood and reviewed^[^^10^^]^, and it represents one of the oldest DNA base excision repair pathways, which constitutes a major area in studies of DNA repair mechanisms and led to the 2015 Nobel Prize in Chemistry.

Charcoal workers with mutant *OGG1* genotypes have higher urinary 8-OHdG content and TM as compared with wild-type genotypes. These results imply that charcoal workers with mutant *OGG1* genotypes are more susceptible to oxidative and genotoxic DNA damage. Since oxidative and genotoxic damage are important considerations in events leading to cancer after carcinogenic/ genotoxicant exposure, precautions should be taken by occupational workers to minimize direct exposure to PAH arising from the incomplete combustion of wood. Accordingly, there is a need to inform these workers about the potential hazards of occupational exposure and should always be provided with appropriate personal protective equipment.

The outcomes of the present study show that there is a significant association of *OGG1* heterozygous (wt/mt) and homozygous (mt/mt) variants with the oxidative and genetic damage as assessed by 8-OHdG level and TM content, respectively in the studied population. These findings suggest that charcoal workers with mutant *OGG1* genotypes are more susceptible to the oxidative and genotoxic DNA damage. However, further studies should be conducted on the DNA repair gene expression and DNA repair capacity with a large sample size.

**Table 4:** Association of *OGG1* genotype with the urinary 8-OHdG content (ng/mg creatinine) and TM (µm)

**OGG1 polymorphism**	**Urinary 8-OHdG**			**TM (µm)**		
	**β** ^a^	**R** ^2^	***p*** ^b^ ** value**	**β** ^a^	**R** ^2^	***p*** ^b^ ** value**
Control group (n = 79)	0.073	0.236	0.025^*^	0.022	0.149	0.000^*^
Exposed group (n = 77)	-0.162	0.603	0.000^*^	-0.168	0.576	0.000^*^

Regression analysis was used to test differences in the urinary 8-OHdG content and TM adjusted for age, consumption habits, and exposure duration. ^*^values are significant. ^a^Unstandardized coefficient; ^b^model *p* value
